# Neuro-ophthalmic Manifestations of Giant Cell Arteritis: A Review

**DOI:** 10.18502/jovr.v20.15248

**Published:** 2025-06-24

**Authors:** Jo-Ann Khoury, Danah Albreiki

**Affiliations:** ^1^Department of Ophthalmology, University of Ottawa, Ottawa, Canada; ^2^King Fahad Medical City, Riyadh, Saudi Arabia

**Keywords:** Giant Cell Arteritis, Neuro-Ophthalmology, Vision Disorders, Visual Symptoms

## Abstract

Giant cell arteritis (GCA) is the most common vasculitis of large and medium vessels affecting adults over the age of 50. Due to its acute ischemic damage through a systemic inflammatory process, GCA is a medical emergency with the risk of permanent vision loss. Therefore, early intervention is critical. Patients often present with well-documented systemic manifestations such as jaw claudication, headache, scalp tenderness, and fatigue. These patients are usually subject to blood tests for inflammatory markers and temporal artery biopsy. However, clinical manifestations vary considerably and may masquerade as neuro-ophthalmic manifestations that are not pathognomonic of GCA. We conducted a review to discuss documented neuro-ophthalmic manifestations and provide insight into the rare presentations to help avoid pitfalls in its diagnosis. Findings from the included articles were sorted into afferent and efferent neuro-ophthalmic manifestations. According to our literature review, the main afferent manifestations documented are ischemic optic neuropathy, retinal artery occlusion, choroidal infarction, ocular ischemic syndrome, orbital inflammatory syndrome, and strokes causing visual field loss. The efferent manifestations include cranial neuropathy (3 ${}^{\text{rd}}$ , 4 ${}^{\text{th}}$ , and 6 ${}^{\text{th}}$ ), extraocular muscle ischemia, and internuclear ophthalmoplegia. Other rare causes are tonic pupil from ciliary ganglion involvement, uveitis, and encephalopathy. If GCA is suspected, in addition to inquiring about systemic symptoms and doing a complete neuro-ophthalmic exam, these patients should be sent for inflammatory serological markers, as well as temporal artery biopsy (TAB). If clinical suspicion remains high, high-dose steroids should be started immediately.

##  INTRODUCTION

Giant cell arteritis (GCA) is a granulomatous vasculitis that typically occurs in adults over iliac arteries. Histopathological studies have shown inflammation to the arterial wall with predominance of CD4+ T lymphocytes and macrophages forming a granulomatous organization.^[2]^ This leads to a loss of vascular smooth muscle cells and elastic fibers, accompanied by an inflammation-induced vascular remodeling. This results in lumen occlusion leading to the ischemic symptoms and signs of GCA,^[2]^ such as the neuro-ophthalmic manifestations discussed in this paper. This vasculitis can lead to visual disturbances caused by ischemic events of the afferent or efferent visual pathway.^[3]^ Given their progressive nature, acute neuro-ophthalmic manifestations of GCA are often seen as emergencies requiring immediate care.

The diagnosis of GCA is typically made using the American College of Rheumatology criteria, which involves clinical systemic symptoms, a positive TAB, and elevated erythrocyte sedimentation rate (ESR), C-reactive protein (CRP), and potentially elevated platelet counts.^[3]^ However, it is important to keep in mind that this disease can present itself in a wide spectrum, and a high index of suspicion is required to reduce the potential of missing this critical disease. High-dose steroid is the standard treatment, usually initiated promptly in a case with high suspicion for GCA. Although glucocorticoids remain the mainstay treatment, administration of tocilizumab has shown promising results in particular cases of GCA.^[4]^

If GCA is suspected, in addition to asking about GCA symptoms and doing a careful and full neuro-ophthalmic exam, these patients should be sent for inflammatory serological markers (looking for relative thrombocytosis and high ESR and CRP) and TAB. If clinical suspicion is high, these patients should be started on high-dose steroids immediately.

##  METHODS

We conducted a systematic review with the objective of having an updated review on neuro-ophthalmic complications of GCA and provide insight into the rare presentations to help avoid pitfalls in its diagnosis. We searched the Ovid MEDLINE database. This search resulted in a total of 808 articles. From this total, 35 articles were included. Criteria for inclusion were articles published from 1990 onwards, a diagnosis of GCA characterized by a positive TAB or American College of Rheumatology criteria, presenting afferent and/or efferent neuro-ophthalmic manifestations being and available in English. The exclusion criteria included articles that did not clearly distinguish between polymyalgia rheumatica (PMR) and GCA, and articles that did not differentiate between neuro-ophthalmic and other manifestations of GCA. We defined neuro-ophthalmic manifestations as complications involving both the visual nervous system and the ophthalmic system. These manifestations were then categorized into afferent and efferent presentations of GCA.

The articles reviewed included afferent manifestations such as ischemic optic neuropathy, transient or permanent retinal arterial occlusion, paracentral acute middle maculopathy (PAMM), choroidal infarction, optic perineuritis (OPN), and stroke. Efferent manifestations included extraocular muscle ischemia, cranial neuropathy, and brain stem stroke (leading to internuclear ophthalmoplegia [INO] or skew deviation, amongst other neurological signs). Among the reviewed articles, there were also rare neuro-ophthalmic manifestations such as tonic pupils, uveitis, and encephalopathy.

To enhance the depth and breadth of our review, we included an additional 24 references. These references were selected to provide historical and seminal context, align with current clinical guidelines, and support specific points by offering detailed explanations of pathophysiology. This comprehensive approach ensures that our review is thorough and reflects the most up-to-date understanding of the neuro-ophthalmic manifestations of GCA.

##  RESULTS AND DISCUSSION 

### Afferent Manifestations

#### Ischemic optic neuropathy (ION)

ION is defined as sudden vision loss caused by the interruption of blood supply to the optic nerve. Interruption of blood flow from the SPCA causes optic nerve head swelling, which is the hallmark of anterior ischemic optic neuropathy (AION).^[5]^ Contrarily, interruption of the pial arteries leads to posterior ischemic optic neuropathy (PION), which does not present with swelling of the optic nerve.^[6]^ When faced with AION, it is crucial to differentiate between an arteritic and a non-arteritic cause. Arteritic AION (AAION) accounts for roughly 5–10% of AION cases, making non-arteritic AION (NAION) the much more common cause of approximately 90–95% of AION cases.^[6]^ AAION accounts for roughly 85% of vision loss cases in GCA, making it the most common cause of vision loss in this vasculitis.^[7]^

In AAION, optic nerve head infarction results from inflammation and subsequent thrombosis of the short posterior ciliary arteries (SPCA).^[6]^ Given its vasculitic etiology, it is often, but not always, associated with systemic symptoms. In NAION, it is believed that the swelling caused by hypoperfusion evokes a compartment syndrome mechanism that compresses the optic nerve axons, thus leading to its compromise. Therefore, associated risk factors include hypertension, diabetes, obstructive sleep apnea, and anatomically crowded optic discs (small cup-to-disc ratio) and the presence of optic nerve head drusen.^[6]^ Differences are also present in the retinal findings. Chalky-white pallid disc edema is a vital feature suggestive of AAION.^[8]^ In contrast, NAION tends to occur in crowded discs and rarely heals with cupping.^[5]^ Rather, diffuse edema with a hyperemic disc followed by pallor without cupping is the hallmark disc appearance following NAION,^[9]^ whereas cupping occurs in 92% of AAION after the swelling resolves.^[10]^ In terms of treatments, AAION management involves urgent corticosteroids, whereas NAION management is mainly focused on mitigating underlying risk factors.^[6]^

In AION, paying attention to clinical features that might suggest an arteritic pathology is of utmost importance. Even in cases where patients are systemically asymptomatic (clinically occult GCA) or serologically normal (serologically occult GCA), noting any of the following features must direct to the possibility of GCA. In cases where patients present with transient monocular vision loss prior to the onset of AION, GCA must be strongly considered,^[11]^ as the cause of such a presentation is rare for NAION.^[12]^ Other features suggesting GCA are advanced age, severe vision loss ( > 20/200), associated cotton wool spots, PAMM or retinal artery occlusion, the asymptomatic contralateral eye with a lack of disc at risk, or a contralateral eye with vasculitic features such as cotton wool spots, even if asymptomatic.^[13]^

**Table 1:** Differences between AAION and NAION^[5–9comma14]^

**Feature**	**AAION**	**NAION**
Etiology	Caused by inflammation of blood vessels (e.g., Giant Cell Arteritis)	Caused by noninflammatory vascular insufficiency
Demographics	Typically, in adults about 60 and older	Usually in middle-aged to older adults (over 50 years)
Symptoms	Systemic: Often present: headaches, scalp tenderness, jaw claudication, polymyalgia rheumatica, constitutional symptoms Vision loss: May be preceded by transient monocular vision loss Usually severe and sudden (often > 20/200)	Generally absent, no systemic symptoms Acute, painless vision loss without preceding symptoms Variable, often less severe, sudden
Retinal findings	Cotton wool spots, retinal artery occlusion, pallid edema	Rarely cotton wool spots, pallid edema not typical
Optic disc appearance	Chalky-white pallid edema, often progresses to optic atrophy with cupping Contralateral eye often asymptomatic with lack of disc at risk, may have vasculitic features such as cotton wool spots	Diffuse edema, hyperemic appearance, rarely pallid Contralateral eye often has crowded disc (small cup-to-disc ratio), no vasculitic features
Investigations	Elevated inflammatory markers (ESR, CRP, PLT) Positive temporal artery biopsy	Normal inflammatory markers Vasculopathic testing may be abnormal for DM, HTN, dyslipidemia, or obstructive sleep apnea Optic disc drusen may be present on autofluorescence, B scan, or EDI OCT RNFL
Treatment	High-dose corticosteroids urgently and tocilizumab	No effective treatment; manage risk factors
Prognosis	Poor if untreated; high risk of vision loss in the other eye	42% improve, up to 30% remain stable, up to 30% worsen. There is also a 15% chance of contralateral eye involvement.^[14]^

#### Retinal artery occlusion

Central retinal artery occlusion (CRAO) is another afferent neuro-ophthalmic manifestation of GCA, leading to severe vision loss. CRAO is divided into two categories: arteritic and non-arteritic. Roughly 95% of CRAO cases are classified as non-arteritic, whereas arteritic causes represent 5% of CRAO cases.^[15]^ Therefore, a stroke workup should be done (including blood pressure and neuroimaging with CT/CTA or MRI/MRA) to assess for infarctions or cardioembolic sources, in addition to treatment of the underlying cause. The arteritic form of CRAO is caused by thrombosis of the central retinal artery (CRA) and typically also involves occlusion of the SPCA. This is explained by the fact that the CRA usually branches off the ophthalmic artery by a common or proximal trunk with the SPCA.^[15]^ Fundoscopy typically reveals a cherry-red spot around the fovea, a pathognomonic finding of retinal artery occlusion due to the retained choroidal perfusion under the retina. This spot may be less evident in cases involving the central retinal and ophthalmic arteries.^[15]^

In a patient 50 years of age or older presenting with vision loss related to CRAO, GCA should always be ruled out. A fundus fluorescein angiography can be performed to assess for underlying choroidal filling delay, indicating PCA involvement, as occlusion of both PCA and CRA in such a patient should raise suspicion for GCA. In cases of CRAO with overlying AION, the diagnosis is GCA until proven otherwise.

Importantly, retinal arterial occlusion may be preceded by amaurosis fugax, caused by transient acute retinal ischemia. These patients should be worked up for both non-arteritic and arteritic causes.^[15]^

**Figure 1:**
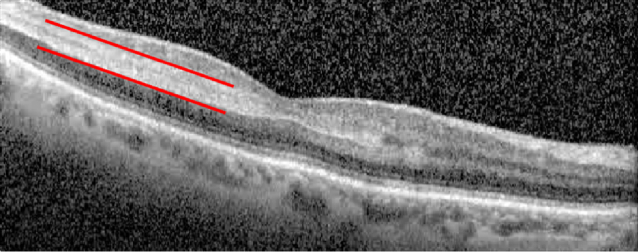
OCT showing hyperreflective bands at the level of the inner nuclear layer of the retina, representative of PAAM.

**Figure 2:**
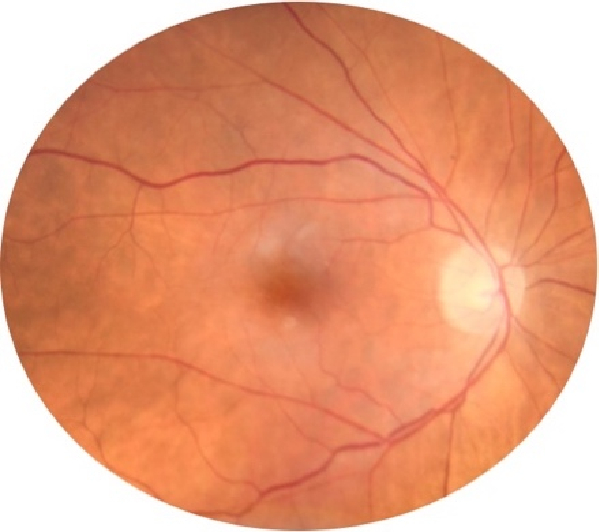
Fundoscopy showing hypopigmented parafoveal lesions representative of PAAM. Colored fundus photo showing the posterior segment of the right eye, just superior to the macular, there are subtle whitish lesions.

Of important note, PAMM, when present in isolation (seen on macular OCT) or more particularly when in association with CRAO or AION, GCA must be highly suspected.^[16]^ PAMM is believed to be ischemia of the middle retinal plexus, exhibiting a hyperreflective band of the inner nuclear and outer plexiform layers on optical coherence tomography of the macula [Figure 1], along with hypopigmented intraretinal parafoveal lesions [Figure 2].^[16]^ This is a more recent OCT sign that should be searched for in all cases of optic nerve or retinal ischemia, as its presence in that association is highly suspicious for GCA.^[17]^

#### Choroidal infarction

A recent study found choroidal infarction to have a 5% incidence rate in patients with GCA.^[18]^ This process is usually associated with the acute phase of GCA-mediated occlusion, which leads to hypoperfusion of the posterior ciliary arteries. On fundoscopy, these can appear as white spots along the retina.^[19]^ As the choroid is the most vascularized layer of the eye, it ensures retinal homeostasis. Therefore, any impact on the retina secondary to choroidal ischemia can lead to vision disturbances.^[20]^

#### Ocular ischemic syndrome

Ocular ischemic syndrome (OIS) is a documented neuro-ophthalmic complication of GCA due to carotid artery occlusion and, consequently, ocular hypoperfusion.

The most common cause of OIS is carotid occlusive disease, commonly related to underlying atherosclerosis.^[21]^ Given this underlying atherosclerotic nature, it usually has a higher incidence in older men.^[22]^ However, OIS has also been documented with occlusive involvement situated in the ophthalmic, central retinal, and posterior ciliary arteries. Often, a healthy anastomotic perfusion may delay clinical symptoms of OIS. More specifically, anastomotic circulation from the circle of Willis will perfuse the ipsilateral ophthalmic artery via anterograde flow, constituting a pathognomonic finding in OIS.^[21]^ Other reported causes include systemic diseases causing vasculitic occlusion, such as GCA and Takayasu's arteritis in younger patients.^[21]^

The clinical presentation can be divided into manifestations of the anterior segment and of the posterior segment. Anterior features include, amongst others, inflammation of the anterior chamber, posterior synechia, corneal edema, iris atrophy, low intraocular pressure, and conjunctival injection. Posterior segment features include, most commonly, narrowed retinal arteries, dilated retinal veins, retinal hemorrhages in the mid periphery, and microaneurysms.^[21]^ In later stages, neovascularization of the iris and posterior segment may ensue causing a presentation of painful neovascular glaucoma.^[21]^

In terms of diagnostic modalities, fluorescein angiography shows a delay in choroidal filling as the most specific feature of OIS.^[21]^ This entails delayed filling that may extend to a duration longer than 1 minute.^[21]^ The most sensitive feature, seen in roughly 95% of cases, is prolonged arteriovenous transit time. More specifically, this is described as a complete retinal venous filling time of over 11 seconds from the initial appearance of retinal arterial dye.^[21]^

#### Orbital inflammatory syndrome (optic perineuritis)

Optic perineuritis (OPN), a subset of orbital inflammatory syndrome, can be a manifestation of GCA. OPN is an inflammation of the optic nerve sheath with multiple potential etiologies. These can be divided into two categories: primary (idiopathic) and secondary. Secondary etiologies include systemic conditions such as infectious, inflammatory, such as GCA and sarcoidosis, lymphoproliferative, demyelinating, and IGG4-related disease.^[23]^

Optic perineuritis likely represents inflammation of the small pial arteries that circumferentially supply the entire orbital portion of the optic nerve. OPN can resemble optic neuritis clinically. In most cases, patients present with vision disturbances and pain on eye movements. As the optic nerve axons are spared, central vision is typically preserved. Unlike optic neuritis, OPN may manifest in association with orbital signs such as ptosis, exophthalmos, and ophthalmoplegia. This is due to its orbital involvement, which is not present in the case of an isolated optic neuritis.^[24]^ OPN can mimic atypical optic neuritis and can present in older adults (40+), requiring medical intervention with steroids to stop its progression. Given its progressive nature and potential association with GCA, OPN must be recognized rapidly to adequately manage and investigate GCA.^[25]^ As both GCA and OPN from other causes are treated with steroids, OPN secondary to GCA may be missed due to early use of corticosteroids.

Diagnosis of OPN is done by MRI using gadolinium and fat suppression of the orbit.^[25]^ Imaging typically shows circumferential enhancement of the intraorbital section of the optic nerve sheath. These present as tram track signs in axial view and donut signs in coronal view. It is important to note that these signs are not specific to OPN and can be seen in any condition affecting the optic nerve sheath, such as optic nerve sheath meningioma. However, the history in the latter is much more chronic, progressive, and typically painless.^[26]^

#### Stroke causing visual defects

Stroke remains a leading cause of disability worldwide. Although it is usually associated with classical etiologies such as atherosclerosis, it has been described in the literature as a rare afferent neuro-ophthalmic complication of GCA. In fact, the incidence of GCA-mediated stroke ranges from 3 to 7%.^[27]^ This can lead to a homonymous visual field deficit, or in more severe bilateral cases, complete blindness due to juxtaposed homonymous hemianopsia, typically from a posterior circulation stroke leading to bilateral occipital lobe infarcts. According to two recent studies, the majority of stroke cases associated with GCA are vertebrobasilar and are more common in men.^[28]^

GCA-associated stroke is ischemic in nature. Macrophages play an important role in GCA as they infiltrate the arterial wall, and through secretion of matrix metalloproteinases and cytokines, there is degradation of the extracellular matrix.^[27]^ The macrophages produce reactive oxygen species, which damage the vascular smooth muscle and endothelium. In addition, vascular endothelial growth factor secreted by the macrophages leads to myofibroblast proliferation and synthesis of extracellular matrix proteins. These events favor intimal thickening and may lead to vessel occlusion.^[27]^

Given its nonspecific features, the diagnosis of GCA in stroke patients may be easily missed unless it is preceded by classical GCA symptoms. Although stroke isn't a common complication of GCA, it has a significant impact on the clinical prognosis of the patient. Therefore, cases that raise suspicion for GCA-associated strokes should prompt immediate treatment and further diagnostic evaluation. Both ESR and CRP tend to be elevated in cases of stroke with underlying GCA. However, these findings are nonspecific and cannot confirm a diagnosis.^[27]^ In an emergency setting, a duplex ultrasonography can aid in diagnosing GCA. A halo sign, produced by a hypoechoic circumferential arterial mural thickening in the cervical arteries, can be the first diagnostic sign of GCA in these cases.^[27]^ TAB remains the standard diagnostic test.

### Efferent Manifestations

#### Diplopia 

Diplopia is a rare complication of GCA with a multifaceted etiology; more commonly, it is transient. It is reported by 1–19% of patients with GCA.^[29]^ Diplopia in GCA may result from a supranuclear, internuclear, or infranuclear cause. Most commonly, the etiology is infranuclear which may be related to cranial nerve (3 ${}^{\text{rd}}$ ,4 ${}^{\text{th}}$ , or 6 ${}^{\text{th}}$ ) or extraocular muscles (EOM) ischemia.^[8]^ Sixth cranial nerve palsy is the most common cause of diplopia in GCA.^[8]^ INO and skew deviation have both been reported to occur in GCA.

Given its nonspecific manifestation, the diagnosis of GCA in an older patient presenting with diplopia poses a serious challenge. However, a study by Ross et al suggested that these patients with underlying GCA and diplopia tend to also present with the concerning systemic manifestations of GCA. This highlights the importance of investigating inflammatory markers and doing a TAB in these patients for whom there is a suspicion of underlying GCA.^[28]^

#### Third nerve palsy

The mechanism of third nerve palsy secondary to CGA seems to be related to microvascular ischemia of the nerve. This can be explained by the fact that GCA affects branches of the posterior cerebral artery, from which the blood supply to the oculomotor nerve arises.^[30]^ Although a third nerve palsy manifestation is generally considered to be related to a posterior communicating artery aneurysm (PCOM) until proven otherwise, one should consider GCA in such cases with patients above 50. A study by Thurtell et al highlighted the importance of early detection of GCA in terms of prognosis for such cases.^[30]^ It was discussed that patients with associated GCA showed rapid improvement of third nerve palsy with early steroid treatment. These patients recovered faster than would be expected for a microvascular third nerve palsy.^[30]^

#### Fourth nerve palsy

The trochlear nerve innervates the superior oblique muscle, responsible for intorsion, abduction, and depression. Therefore, a trochlear palsy manifests as an ipsilateral hypertropia and excyclotorsion.^[31]^ Vascular causes are among its possible etiologies.^[31]^ It is important to note that this can present similarly to skew deviation. In contrast to fourth nerve palsy, skew deviation is usually secondary to brainstem stroke and presents with incyclotorsion of the hypertropic eye.^[18]^

#### Sixth nerve palsy

Abducens palsy presents as binocular horizontal diplopia due to a lateral rectus paresis.^[32]^ A case report presented as isolated bilateral abducens nerve palsy due to underlying GCA.^[32]^

#### Internuclear ophthalmoplegia

Internuclear ophthalmoplegia (INO) is an ocular movement disorder that manifests as an inability to adduct the eye, or a slow adducting saccade, along with abducting nystagmus in the contralateral eye.^[33]^ It is due to damage to the medial longitudinal fasciculus (MLF) that can be affected by ischemic, inflammatory, demyelinating, or neoplastic lesions in the pons or in the midbrain. Thus, INO can be caused by GCA-mediated ocular muscle ischemia, as it affects the basilar and ophthalmic arteries.^[34]^

### Rare Manifestations

#### Tonic pupil

Despite the propensity for vascular occlusions in the eye in patients with GCA, the tonic pupil has rarely been reported as one of its neuro-ophthalmic manifestations, presumably due to the anastomotic pattern of the arteries supplying the ciliary ganglion.^[35]^ In the articles yielded from our search, light-near dissociation (LND) was not reported in isolation but rather was accompanied by an optic neuropathy^[35]^ or following systemic symptoms of GCA.^[36]^ These patients usually present with light near dissociation and vermiform contraction of the iris sphincter muscle. Unlike the documented acute nature of LND in GCA, this is usually a chronic finding in tonic pupil. This can be explained by the distinct mechanisms by which GCA and tonic pupil lead to LND. In the latter, LND develops following the chronic process of denervation and subsequent aberrant regeneration of the postganglionic parasympathetic fibers.^[35]^ Conversely, LND seems to manifest earlier in GCA due to the acute ischemic damage to the afferent visual pathways involved in the pupillary light reflex. In short, the pathophysiological mechanism of GCA most likely involves vasculitis-mediated ischemic damage to the parasympathetic ciliary ganglion.^[35]^

#### Encephalopathy

This review included a case of encephalopathy in a patient with GCA. This patient presented with general malaise and cognitive changes such as confusion, obsessive behavior, and being easily startled by noises.^[37]^ Another article reported a similar case of an elderly patient known for GCA presenting with a new onset of focal neurologic symptoms. This was categorized as a posterior reversible encephalopathy due to vasogenic edema in the brain. It was suggested that a concomitant inflammatory state can catalyze the evolution of cerebral vasogenic edema, eventually leading to a form of cytotoxic edema. Vasculitis, such as GCA, constitutes a prime example of diseases causing an inflammatory state.^[38]^ Although encephalopathy is not a well-documented complication of GCA, any suspicion of this in a patient should prompt MRI imaging and treatment with glucocorticoids.

#### Uveitis 

Uveitis is another rare manifestation of GCA. In fact, its relevance as a true ocular manifestation secondary to GCA has been argued as a chance association by some studies.^[39]^ According to our search, there are very few reported cases of uveitis presumably secondary to biopsy-proven GCA. The article included in our search presented a patient with a unilateral anterior uveitis that was diagnosed only three weeks prior to an official diagnosis of GCA. However, this patient had initially presented with systemic manifestations such as weight loss, headache, and vision loss. Eventually, the patient developed the classical symptoms of GCA, such as jaw claudication and scalp tenderness. Although these were relieved by steroid treatment, there was no improvement in his vision.^[40]^ Despite the unusual association of anterior uveitis with GCA, physicians should rule out vasculitis in similarly presenting cases of uveitis, which would be challenging if this precedes the systemic symptoms of GCA.^[41]^

### Diagnosis

#### Clinical presentation

In any patient with suspected GCA, a detailed history taking and neuro-ophthalmic examination should be performed. Clinically, the most common symptoms of GCA are a new onset of headache, scalp tenderness, visual disturbances, and jaw claudication being the most specific one.^[42]^ Temporal arteries may be thickened and tender to palpation in up to 60% of cases.^[43]^ The temporal arteries may also have a decreased pulse strength. Signs such as pallid edema of the optic nerve head and cotton wool spots should raise suspicion for optic nerve involvement or retinal ischemia.^[43]^ As mentioned earlier, diffuse white lesions are associated with choroidal infarction.

Transient visual loss can progress to become bilateral and permanent within 14 days in untreated cases.^[44]^ An ophthalmic exam would also detect some of the aforementioned efferent complications of GCA, such as restricted eye movements. It is crucial to mention that patients with GCA may present with ocular manifestations but not have any additional systemic symptoms. This is known as clinically occult GCA and can occur in up to 21% of cases.^[45]^

#### Serology

The laboratory values assessed in the diagnosis of GCA are ESR, CRP, and the platelet count. An elevated ESR greater than 50 mm/hour is part of the American College of Rheumatology criteria for GCA. However, it has been reported that ESR can occasionally be normal in patients with a biopsy-proven case of GCA.^[8]^ Additionally, ESR may be elevated by other diseases of an inflammatory nature and may be lowered by the use of steroids for immunosuppression.^[46]^ The CRP is often used in conjunction with the ESR to diagnose GCA. Unlike the ESR, CRP is not as influenced by age and hematological factors, and it seems to have a higher accuracy for diagnosing GCA. A study demonstrated a 100% sensitivity and 97% specificity for an elevated CRP in diagnosing GCA.^[8]^ Studies have also shown the use of elevated platelet count as a good predictor of GCA. However, this did not surpass ESR and CRP in their predictive ability for GCA.^[47]^

One should be aware of serologically occult GCA in which patients present with visual and/or systemic GCA symptoms, normal serology, yet a positive TAB. This is important to keep in mind as a normal serology does not rule out GCA.

#### Imaging

Fluorescein angiography and neuroimaging can also aid in the diagnosis of GCA. In suspected cases of choroidal or retinal hypoperfusion secondary to GCA, fluorescein angiography can demonstrate a delay in perfusion to those areas.^[29,45]^ This, alongside symptoms of GCA, should prompt immediate treatment. Neuroimaging is helpful for the diagnosis of orbital and infraorbital nerve sheath enhancement, such as seen in OPN. More specifically, an MRI using gadolinium and fat suppression is used for these suspected cases.^[25]^ Lastly, ultrasonography can be used to diagnose GCA. A halo sign in the temporal arteries is predictive of GCA. This is produced by a hypoechoic circumferential arterial mural thickening.^[27]^

#### Temporal artery biopsy

Temporal artery biopsy (TAB) is the gold standard for diagnosing GCA. It should be obtained as early as possible in cases with suspicion of GCA, without impeding the start of steroid treatment. A positive TAB is characterized by mononuclear cell infiltration or granulomatous inflammation and multinucleated cells.^[48]^ Although the TAB has a high specificity for diagnosing GCA, its sensitivity is often much lower as a consequence of poor sampling and skip lesions. Therefore, it should be obtained from the most symptomatic site with segments long enough to account for these skip lesions and for a 10% tissue shrinkage during fixation.^[49]^ In some institutes, a bilateral temporal artery with sufficient length can be done to reduce the risk of missing contralateral positivity, as reported.^[50]^

### Practical Recommendations for the Diagnosis of GCA

#### History

In any suspected case of GCA, a complete and detailed history should be obtained, particularly inquiring about new onset of headache, scalp tenderness, visual disturbances, and most specifically jaw claudication, in addition to constitutional symptoms (fatigue, fever, night sweats, weight loss) and PMR symptoms.^[42]^ It is important to note that there is clinically occult GCA, in which case the patient presents with visual but not systemic symptoms, and has high inflammatory markers and a positive TAB. This is important to keep in mind as a lack of systemic symptoms does not rule out GCA.^[45]^

#### Exam

In any suspected case of GCA, a complete and detailed neuro-ophthalmic exam should be performed. Fundus exam should be done to assess for signs of pallid edema of the optic nerve head, and cotton wool spots. On the efferent exam, clinicians should look for restriction of extraocular eye movements and ocular misalignment. More generally, clinicians can also examine the temporal arteries, which may present with thickening, tenderness, or decreased pulse strength.^[43]^

#### Investigations

ESR, CRP, and platelet levels should be part of the initial workup. Although an elevated ESR is an American College of Rheumatology criterion for GCA diagnosis, it can be normal in some patients. An elevated CRP and ESR are highly sensitive. Relative thrombocytosis can support the diagnosis of GCA.^[8,43]^

The gold standard for diagnosing GCA remains TAB. Clinicians should consider long-segment TAB in order to account for possible skip lesions and increase the procedure's sensitivity for a GCA diagnosis.^[27]^

#### Additional imaging 

In cases where TAB is not acutely accessible, clinicians can consider fluorescein angiography (to detect delays in choroidal filling)^[21]^ or ultrasonography (looking for halo sign) to support the diagnosis of GCA.^[27]^

If PAMM is present in isolation in a patient suspected of having GCA or if present with AION, then GCA should be highly suspected.^[16]^ Delayed choroidal perfusion might be present.

### Management

The standard initial treatment of GCA is corticosteroids. Given the rapidly progressing nature of GCA, high-dose corticosteroids should be started immediately in a patient with suspected GCA. They should not be delayed by other investigations such as TAB or serology.^[51]^ High-dose corticosteroids should be initiated immediately upon clinical suspicion of GCA to prevent irreversible vision loss and other complications such as stroke and aortitis. Typically, this involves a starting dose of 1 mg/kg/day. Alternatively, intravenous methylprednisone (1000 mg/day for three days) is recommended in patients with acute vision loss or severe ischemic complications. Monitoring of side effects in steroid therapy is of utmost importance. The dosage of corticosteroids can be tapered gradually and slowly based on clinical response and on normalization of inflammatory markers such as ESR and CRP, as well as platelet count.^[52]^ The utility of glucocorticoids for preventing vision loss has been shown in observational data. In a study of 144 patients with biopsy-proven GCA, no deterioration of vision was seen in patients without vision loss at presentation. Thus, in cases of intact vision at presentation, management with glucocorticoids seemed to reduce the risk of vision loss to less than 1%.^[44]^ In addition, a small study reported that roughly 9% of patients will experience involvement of the fellow eye following corticosteroid therapy, whereas this may be experienced in 20–62% of untreated patients.^[53]^ In cases of resistance to steroid therapy, especially with a negative TAB, the diagnosis of GCA should be revisited, keeping in mind that a small subset of patients may have aggressive and progressive steroid-refractory GCA.

#### Steroid-sparing agents

Glucocorticoid-mediated adverse effects such as diabetes, osteoporosis, glaucoma, and hypertension are well documented. In cases with such complications or in cases of glucocorticoid-refractory GCA, steroid-sparing agents may be used.^[4]^ Common steroid-sparing agents are methotrexate and tocilizumab.

Methotrexate, a disease-modifying anti-rheumatic drug (DMARD) used in a broad spectrum of systemic inflammatory diseases, has shown modest effects as a treatment for GCA. A meta-analysis reported lower relapse rates and higher rates of glucocorticoid-free remission.^[54]^ Overall, methotrexate has been shown to suppress disease progression when used in low doses and in combination with glucocorticoids^[1]^ and decrease the rate of relapse when compared to treatment with glucocorticoids alone.^[55]^

Tocilizumab is a humanized anti-interleukin-6, thought to decrease the inflammation cascade.^[3]^ It is found to be effective as an adjunct to glucocorticoids, aiding in faster taper and showing promising results as a therapy for refractory or relapsing disease. However, it is not reported to be effective when used in flares.^[4]^ Tocilizumab is relatively new in the GCA treatment and is showing promising results. Reports have shown that tocilizumab leads to a fast and preserved improvement both clinically and serologically.[56–59] A recent study also showed greater rates of steroid taper and remission compared to a placebo group.^[60]^ As tocilizumab is associated with higher risk of infection, gastric perforation, and diverticulitis, patients using it should be routinely monitored with lab screenings such as liver function tests, platelets, and neutrophils.^[4]^

### Practical Recommendations for the Management of GCA 

#### Corticosteroid therapy

Initiation: Upon clinical suspicion of GCA, immediate treatment should be started to prevent irreversible vision loss and complications. TAB or serology must not be delayed.

Dosing: Typically, a starting dose of 1 mg/kg/day of oral prednisone is recommended. For patients with acute vision loss or severe ischemic complications, clinicians should consider intravenous methylprednisolone (1000 mg/day for three days).^[52]^

#### Resistance to steroid therapy

Reassess diagnosis if resistance occurs, especially with a negative TAB. Consider steroid-refractory GCA.

In patients with relapsing or refractory GCA or those who develop significant side effects from corticosteroids, clinicians should consider steroid-sparing agents.^[4]^

#### Use of steroid-sparing agents

Methotrexate: Consider use for glucocorticoid adverse effects or refractory cases. It can reduce relapse rates and aid in glucocorticoid-free remission.^[55]^

Tocilizumab: Consider use for refractory or relapsing disease. However, clinicians should refrain from using it during flares as its effectiveness has not been demonstrated in the literature for flares. Routinely monitor with laboratory testing for liver function tests, platelet counts, and neutrophil levels, due to the risk of infection, gastric perforation, and diverticulitis.^[4]^

#### Patient education and follow-up

Clinicians should educate patients about the potential side effects of treatments and the importance of adherence to therapy. Regular follow-up appointments are essential to adjust treatment plans based on clinical response and to manage any adverse effects.

##  SUMMARY

Given that, at times, GCA symptoms are subtle and often masquerade as common systemic symptoms, early diagnosis can be challenging. Therefore, maintaining a high index of suspicion for GCA in patients who present with these neuro-ophthalmic manifestations is of paramount importance in clinical practice. It is often assumed that patients with GCA always present with systemic manifestations such as headache, fever, jaw claudication, and scalp tenderness, and that in the absence of these symptoms, GCA can be ruled out. However, this may lead to cases of misdiagnosis and delayed treatment. Early diagnosis and prompt start of treatment are key to vision loss prevention in patients with GCA. Therefore, clinicians must immediately begin treatment and investigation for the vasculitis, considering the possible associated neuro-ophthalmic manifestations mentioned in this paper. Afferent neuro-ophthalmic manifestations are the most reported. Among these, arteritic AION is the most documented. Efferent neuro-ophthalmic manifestations usually involve the EOMs. Diplopia due to infranuclear causes, such as cranial neuropathies, was the most reported in this category. In terms of rare complications, this review summarized documented cases of stroke, uveitis, and encephalopathies. Although it is not possible to cover the full spectrum of possible rare and nonspecific complications, clinicians should consider the ischemic and inflammatory nature of GCA when evaluating a potential association with a rare presentation. Given the clinically and serologically occult entities of GCA, patients over 50 presenting with a history of vision loss and any of the neuro-ophthalmic complications mentioned must raise suspicion for this vasculitis. In these cases, patients should be started immediately on high-dose steroids, followed by a TAB and serology to confirm GCA.

##  Financial Support and Sponsorship

None.

##  Conflicts of Interest

None.
